# Hyperosmolarity Triggers the Warburg Effect in Chinese Hamster Ovary Cells and Reveals a Reduced Mitochondria Horsepower

**DOI:** 10.3390/metabo11060344

**Published:** 2021-05-26

**Authors:** Jorgelindo da Veiga Moreira, Lenny De Staercke, Pablo César Martínez-Basilio, Sandrine Gauthier-Thibodeau, Léa Montégut, Laurent Schwartz, Mario Jolicoeur

**Affiliations:** 1 Research Laboratory in Applied Metabolic Engineering, Department of Chemical Engineering, École Polytechnique de Montréal, P.O. Box 6079, Centre-Ville Station, Montréal, QC H3C 3A7, Canada; jorgelindo.daveiga@polymtl.ca (J.d.V.M.); Lenny.DeStaercke@ugent.be (L.D.S.); pablo1903.mb@gmail.com (P.C.M.-B.); sandrine.gauthier-thibodeau@polymtl.ca (S.G.-T.); montegut.lea@gmail.com (L.M.); 2 Assistance Publique des Hôpitaux de Paris, Avenue Victoria, 75003 Paris, France; dr.laurent.schwartz@gmail.com

**Keywords:** CHO cells, Warburg effect, hyperosmolarity, lipoic acid, hydroxycitrate, mitochondrial hyperfusion

## Abstract

Tumor cells are known to favor a glycolytic metabolism over oxidative phosphorylation (OxPhos), which takes place in mitochondria, to produce the energy and building blocks essential for cell maintenance and cell growth. This phenotypic property of tumor cells gives them several advantages over normal cells and is known as the Warburg effect. Tumors can be treated as a metabolic disease by targeting their bioenergetics capacity. Alpha-lipoic acid (ALA) and calcium hydroxycitrate (HCA) are two drugs known to target the Warburg effect in tumor cells and hence induce the mitochondria for ATP production. However, tumor cells, known to have an increased flux through glycolysis, are not able to handle the activation of their mitochondria by drugs or any other condition, leading to decoupling of gene regulation. In this study, these drug effects were studied by mimicking an inflammatory condition through the imposition of a hyperosmotic condition in Chinese hamster ovary (CHO) cells, which behave similarly to tumor cells. Indeed, CHO cells grown in high osmolarity conditions, using 200 mM mannitol, showed a pronounced Warburg effect phenotype. Our results show that hyperosmolar conditions triggered high-throughput glycolysis and enhanced glutaminolysis in CHO cells, such as during cancer cell proliferation in inflammatory tissue. Finally, we found that the hyperosmolar condition was correlated with increased mitochondrial membrane potential (ΔΨm) but mitochondrial horsepower seemed to vanish (h = Δp/ΔΨm), which may be explained by mitochondrial hyperfusion.

## 1. Introduction

There are multiple and reproducible reports in the literature suggesting that hyperosmolarity and inflammation are closely related, if not synonymous. Hyperosmolarity can cause inflammation [1,2,3,4,5,6,7,8,9], and it has been reported in several inflammatory diseases, such as Crohn’s disease and ulcerative colitis [10], inflammatory bowel disease of the newborn and neonatal necrotizing enterocolitis [9], and inflammatory pleural effusion [11]. Any chemical providing sufficient osmolarity can induce an inflammation [1,8,9]. Inflammation has many known tumor-promoting effects, such as the support of cell proliferation and survival of malignant cells, the promotion of angiogenesis and metastasis, the subversion of adaptive immune responses, and the alternation of responses to hormones and chemotherapeutic agents [12,13]. The same osmolarity in chemically unrelated molecules, such as alanine, sodium chloride, or mannitol, has been shown to induce similar pro-inflammatory cytokine secretion [14]. Inflammation is thus widely recognized as a key risk factor for cancer [15]. Indeed, hyperosmolarity has also been measured in tumors from a high interstitial protein concentration [16,17,18].

In 1925, the German biochemist Otto Heinrich Warburg described in his seminal article how cancer cells mainly consume glucose and produce large amounts of lactate, even in the presence of oxygen [19]. Referred as the “Warburg effect”, these observations allowed him to hypothesize the existence of a mitochondrial deficiency in cancer cells [20]. Then, in the absence of respiratory mitochondria, which are the powerhouse of eukaryotic cells, ATP production efficiency is much lower and may be compensated by higher ATP turnover through aerobic glycolysis [21,22]. This is a marker of a reduced mitochondria membrane potential (ΔΨm) such as in cancer cells [20,21]. Another key feature of the cancer metabolism is glutaminolysis [23,24,25,26], which is now being targeted to treat cancer [23,27,28,29,30,31,32]. In addition to an aerobic glycolytic metabolism, there is now mounting evidence indicating that altered glutamine metabolism in cancer cells has critical roles in supporting macromolecule biosynthesis, regulating signaling pathways, and maintaining redox homeostasis, all of which contribute to cancer cell proliferation and survival [27,33,34,35]. Glutaminolysis is thus now seen as another hallmark of cancer [26], together with aerobic glycolysis [21].

Although inflammation is a key risk factor for carcinogenesis, the link between inflammation and cancer cell metabolism is poorly described. Therefore, the goal of this study was to assess the impact of osmotic pressure on both the Warburg effect and glutaminolysis. With mannitol as an osmotic agent, we compared the sole osmotic effect to that obtained when applying the METABLOC drugs combination previously proposed as a metabolic therapy of cancer [36]. Chinese hamster ovary (CHO) cells were selected as the model biosystem since they exhibit the specific traits of most cancer cells, such as the Warburg effect phenotype and infinite growth [37,38]. The METABLOC combination includes alpha-lipoic acid (ALA) and calcium hydroxycitrate (HCA). ALA, which is being used to treat diabetes [39,40,41,42], activates pyruvate dehydrogenase (PDH) by being a potential inhibitor of pyruvate dehydrogenase kinase (PDK), which results in an increased amount of pyruvate entering the tricarboxylic acid cycle (TCA). ALA therefore potentially limits aerobic glycolysis (i.e., the Warburg effect) and, thus, lactate formation. Moreover, there are at least five currently known ALA-using enzyme complexes in mammals. These are pyruvate dehydrogenase (PDH), alpha-ketoglutarate dehydrogenase (KGDH), branched chain alpha-keto acid dehydrogenase, oxoadipate dehydrogenase, and the glycine cleavage system [5,43,44,45]. Furthermore, ALA has also been shown to confer an osmotic protective effect to red blood cells [46]. Calcium hydroxycitrate in its turn is a potential inhibitor of ATP citrate lyase (ACL), an enzyme responsible for the synthesis of cytosolic acetyl coenzyme A (acetyl-CoA) [47]. This drug has also been reported to stimulate autophagy in cancer cells in vitro [48,49] and both effects are finally associated with decreased lipid production, a crucial factor to limit high cell proliferation of tumor cells. The effects of hyperosmolarity, in the presence or absence of drugs, on CHO cells, the Warburg effect, and glutaminolysis, as well as on mitochondrial activity, are here described.

## 2. Results

Two drugs, alpha-lipoic acid (ALA) and calcium hydroxycitrate (HCA), known to target the Warburg effect in tumor cells [50] were assessed in CHO cell cultures. Lipoic acid activates pyruvate dehydrogenase (PDH) by being a potential inhibitor of pyruvate dehydrogenase kinase (PDK), which results in an increased amount of pyruvate. Pyruvate is then the first intermediate of the tricarboxylic acid cycle (TCA), thereby pulling on glycolysis flux. Calcium hydroxycitrate in its turn is a potential inhibitor of ATP citrate lyase (ACL) and thus is expected to decrease fluxes to lipid production, thus limiting a crucial anabolic pathway for highly proliferative tumor cells. We first examined the effect of high osmolarity, both with and without the use of the cocktail of two drugs, on the cell global behavior in order to then focus on the cell cycle distribution and, finally, on the cell mitochondrial activity.

### 2.1. High Osmolarity Condition Affects CHO Cell Growth Rate but Maintains Viability

A first series of experiments was conducted to characterize CHO cell behavior when sub-cultured in high osmolarity media. CHO cells were thawed, acclimated for sufficient passages until a stable reproducible specific growth rate was observed, and then grown in media supplemented with mannitol. Cells were cultured for five successive passages (P1–P5) in a 300 mOsm medium (control), as well as in media at 400 and 500 mOsm, by adding, respectively, mannitol at 100 Mm and 200 mM final concentrations. Increasing medium osmolality at 400 mOsm reduced slightly but reproducibly the specific growth rate of CHO cells (Figure S1A). Having a strong but non-lethal effect, the 200 mM mannitol addition treatment was selected and further studied (see Section 2.2).

**Figure 1:**
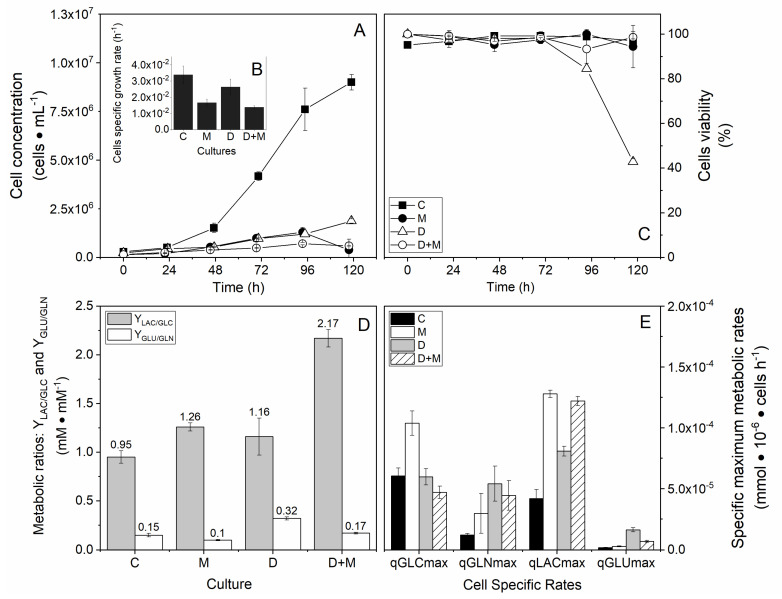
Effects of hyperosmotic culture conditions and drugs combinations on cell growth and metabolic phenotype. Experimental results for the shaken flask batch cultures for control conditions (filled squares, C), high osmolarity conditions induced with 200 mM D-mannitol (filled circles, M), control conditions with the addition of 8 μM alpha-lipoic acid (ALA) and 300 μM calcium hydroxycitrate (HCA) (empty triangles, D), and high osmolarity conditions with the addition of 8 μM ALA and 300 μM HCA (empty circles, D + M). (**A**,**B**) Batch cell concentration and cell growth rate were determined. Cell growth rates were calculated from the maximum growth rate phase in each condition. Cell growths were highly inhibited under the high osmolarity condition and/or with the drugs combinations compared to the control condition. This is highlighted by the cell-specific growth rate, which was higher in the control condition. (**C**) Viable cell density. Cell viabilities were flat and quite similar for all conditions, except for the drugs combination condition where cell viability fell suddenly after 70 h of culture and reached 43% at the end of the culture. (**D**) Metabolic ratios of lactate per mole of glucose consumed (Y_LAC/GLC_, grey bars) and glutamate per mole of glutamine consumed (Y_GLU/GLN_, grey bars) were set. Lactate yield was higher in the D, M, and D + M conditions compared to control. Glutamate yield was higher in the D condition but relatively close to control for the M and D + M culture conditions. (**E**) Specific maximum metabolic rates for each metabolite. Glucose consumption rate was higher under high osmolarity. Glutamine consumption was higher in drugs and/or mannitol compared to control. The same was true for the lactate production rate, which was found to be much more pronounced in drugs and/or mannitol. Glutamate production rate was higher in the D and D + M conditions, which are markers of reduced glutaminolytic rates.

A second series of experiments was then conducted with P5 cells to follow cell growth and carbon fluxes in a control culture (C—filled square), under hypertonic conditions (M—filled circle), with lipoic acid and hydroxycitrate combination drugs (D—empty triangle), and with both drugs and under a mannitol condition (D + M—empty circle). Hyperosmolarity only and drugs combinations drastically decreased cell growth even if initial exponential growth can be observed (Figure 1A,B). A high osmolarity (200 mM mannitol/500 mOsm) culture condition resulted in a significant decrease of the cell division rate to half of its value (control), with a cell-specific growth rate decreasing from 0.03 ± 0.010 to 0.017 ± 0.002 h^−1^, which correspond to an average cell time division (td) between 20.49 ± 2.85 and 41.57 ± 4.84 h (Figure 1A,B). Addition of lipoic acid and hydroxycitrate drugs also affected cell division but to a lower extent than high osmolarity, with a cell-specific growth rate down to 0.026 ± 0.005 h^−1^ and a td of 26.33 ± 4.12 h. However, adding the drugs to the 200 mM mannitol culture further decreased cell growth, with a cell-specific growth rate down to 0.014 ± 0.001 h^−1^ and a td of 50.23 ± 3.25 h. Cell viability stayed high (>95%) except in the culture with the two drugs only, where it declined from 72 h.

Taken alone, both ALA and HCA showed no effects on CHO cell growth rate but resulted in lower final cell counts, while ALA maintained cell viability and HCA caused a decrease of cell viability (Figure S2).

### 2.2. High Osmolarity Condition Favors Glycolytic and Glutaminolytic Phenotypes

The macroscopic biomarkers Y_LAC/GLC_ and Y_GLU/GLN_ for the 200 mM mannitol culture confirmed clear glycolytic and glutaminolytic phenotypes (Figure 1D,E). Y_LAC/GLC_ of 1.26 ± 0.042 and Y_GLU/GLN_ of 0.10 ± 0.0075 were obtained in the mannitol culture compared to respective values of 0.95 ± 0.063 and 0.15 ± 0.019 for the control. Results were similar to those for the successive passage cultures (Figure S1). By only adding the two drugs to the culture, a Y_LAC/GLC_ ratio of 1.16 ± 0.19 was reached, while, in parallel, the Y_GLU/GLN_ ratio increased at twice the value of the control (0.32 ± 0.016 versus 0.11 ± 0.003) (Figure 1D). This result suggests that a larger proportion of the glutamine metabolized was being released by the cells as glutamate (Figure S3C,D). Interestingly, adding the drugs to the mannitol culture led to a high Y_LAC/GLC_ ratio with a value of 2.17 ± 0.26, meaning that, theoretically, all glucose and possibly some other carbon sources were redirected towards lactate release, while a similar Y_GLU/GLN_ ratio (0.1700 ± 0.0009) to that of the control (0.1500 ± 0.0019) was observed (Figure S3A,B). The effect of adding lipoic acid and hydroxycitrate drugs at high osmolarity thus amplifies a glycolytic phenotype but cancels the increase of the glutaminolytic phenotype induced at high osmolarity (Figure 1E).

### 2.3. Cell Cycle Distribution and Early Effect of Hyperosmolarity and Drugs Combination

#### 2.3.1. Cell Cycle Distribution

**Figure 2:**
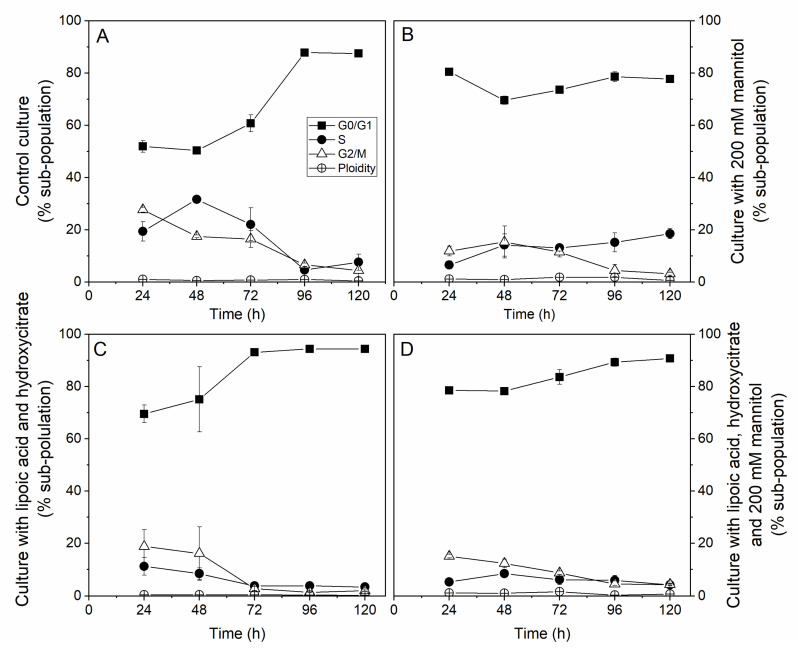
Effects of hyperosmotic culture conditions combined with lipoic acid and hydroxycitrate drugs on CHO cell cycle distribution. Experimental results for the shaken flask batch cultures in the GO/G1 phase (filled squares), the S phase (filled circles), the G2/M phase (empty triangles), and in the ploidy state of cells (empty circles) (n = 30) in: (**A**) control conditions; (**B**) high osmolarity conditions induced with 200 mM D-mannitol; (**C**) control conditions with the addition of 8 μM alpha-lipoic acid (ALA) and 300 μM calcium hydroxycitrate (HCA); and (**D**) high osmolarity conditions with the addition of 8 μM ALA and 300 μM HCA.

The cell populations’ DNA content was stained with DAPI and observed by flow cytometry to determine the treatments effect on cell cycle distribution. The addition of mannitol as well as the two drugs was found to decrease the proportion of cells in the S phase compared to the control (Figure 2A,B). This result is, however, partially in agreement with the respective cell-specific growth rates. With the higher cell-specific growth rate (0.03 ± 0.01 h^−1^), the control culture also exhibited a higher dynamic time profile for the cell sub-populations, with proportions reaching 32% of cells in the S phase at day 2 at the mid-exponential growth phase and decreasing by around 6% from day 4 (plateau growth phase). During the cell exponential growth phase (1–3 days), 50% of the cells were in the G0–G1 phase. However, adding mannitol and drugs, whether in combination or not and which resulted in lower cell-specific growth rates, was found to dampen the dynamic evolution of the cells’ sub-populations, with lower proportions of cells in the S phase and over 70% of the cells being in the G0–G1 phase during the cultures (Figure 2C,D). Adding mannitol resulted in lower sub-populations in the S and G2-M phases at day one with 7% and 12%, respectively. However, the S phase sub-population then slowly but stably increased to 18%, which was contrast with the strong decrease observed in the control culture and those with the drugs. Inoculating in a hyperosmotic medium resulted in a high cell population in the G0–G1 phase (80%) at day one, which decreased to 70% on day two and then increased and reached a plateau at 79% from day 4. In comparison, the cell population in the control culture in the G2-M phase regularly decreased from 28% to 4%, and the G0–G1 cell population followed a cell growth profile, reaching a plateau at 88% from day 4. With lipoic acid and hydroxycitrate, in the absence of mannitol, a clear decrease of the cell population in the S phase from 11 to 4% (from day 3) was observed, with 94% of the cells in the G0–G1 phase from day 3. However, combining the two drugs with mannitol showed a stabilization of an S population between 5–8%. However, the cell population in the G0–G1 phase increased from 79 (day 1) to 91%, a plateau value reached as early as day 3. Therefore, in the presence of the drugs, the trend of the G0–G1 sub-population was similar to that of the control culture but at higher levels. The addition of 200 mM mannitol further increased the G0–G1 sub-population during cell growth. Notably, the reduction of the cell growth rate at high osmolarity did not result in polyploid cells, i.e., with more than one DNA molecule. The level of abnormal polyploid cells was at the noise level with a maximum of 1.84% of polyploid cells in the 200 mM mannitol culture, 1.53% when combining the two drugs to mannitol, 0.43% for the drugs only, and 0.98% for the control culture.

#### 2.3.2. Early Events Following Drugs and Mannitol Addition

**Figure 3:**
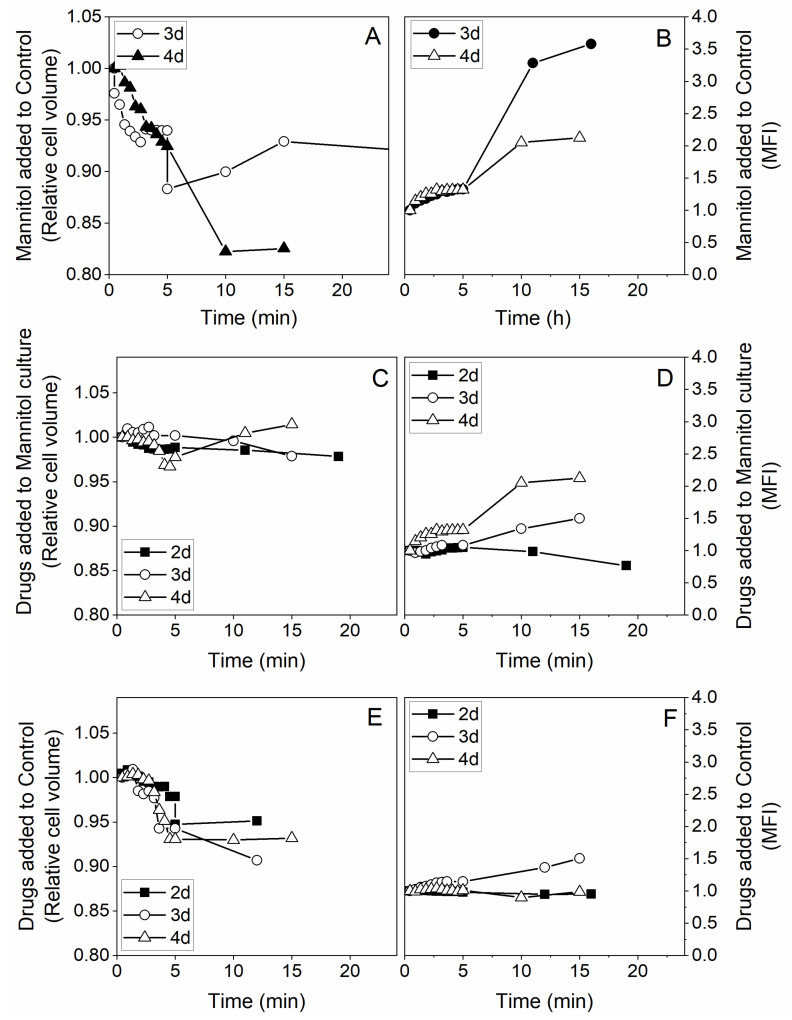
Early events for the effects of adding hyperosmotic culture conditions combined with lipoic acid and hydroxycitrate drugs. Relative cell volume and mean fluorescence intensity (MFI) monitoring for cells after two days of culture (filled squares), three days of culture (empty circles) and four days of culture (empty triangles). (**A**) Relative cell volume evolution upon addition of 200 mM final concentration of mannitol to cell culture. (**B**) MFI of mitochondrial staining with rhodamine 123 upon mannitol addition to cell culture. (**C**) Relative cell volume for mannitol and combination of ALA and HCA drugs. (**D**) MFI monitoring of drugs and mannitol cultures. (**E**) Relative cell volume variation upon ALA and HCA drugs addition to cell cultures. (**F**) MFI monitoring after drugs addition to control condition. All the figure values are relative units as described in the main text.

We then looked at the early effects of adding the two drugs and mannitol on the cell size by monitoring the forward scattered signal (FSC) over time in flow cytometry measurements of cells during the exponential growth phase (i.e., from day 2 to 4). The cell viability was higher than 98% in all samples analyzed. The sudden addition of mannitol 200 mM to a control culture caused a rapid cell size reduction of 6 to 8% with signs of stabilizing after 5 min (Figure 3A). Addition of lipoic acid and hydroxycitrate drugs to either the control culture or the 200 mM mannitol culture also caused a rapid cell size reduction, but to a lesser extent and within a 2 to 7% decrease. These ranges of cell size decrease correspond to a cell volume decrease of 3 to 12% for decreases in cell diameter of 2 and 8% respectively. As a complement to water loss, it is clear the cells did fully compensate, with the uptake of osmolytes, the 67% increase of the medium osmotic pressure that resulted from adding 200 mM mannitol. Although a cell size reduction from mannitol addition was expected, the effect of adding the drugs was less obvious and required analysis on the metabolic side (Figure 3C,E). We thus also looked at the effects on the cell mitochondrial activity.

Cell mitochondrial activity level was also monitored by flow cytometry along with the cell size measurement. Cells were stained with rhodamine 123 (Rh123) for analysis of early events in mitochondrial membrane polarization (H + gradient). The addition of mannitol caused a 20 to 30% increase, respectively, of the mitochondrial membrane potential within the 5 min of monitoring (Figure 3B). The addition of the drugs to the control culture caused a 5 to 30% increase of the rhodamine 123 total fluorescence per cell, while a 10% increase was observed for drugs added to the 200 mM mannitol culture (Figure 3D,F). The cells thus exhibited a rapid metabolic reaction to perturbations generated both from mannitol and drugs addition, but there was no additive effect observed. These results are further discussed together with long-term cell reactions below.

#### 2.3.3. Long-Term Effects on Cell Mitochondrial Activity

##### Membrane Potential

**Figure 4:**
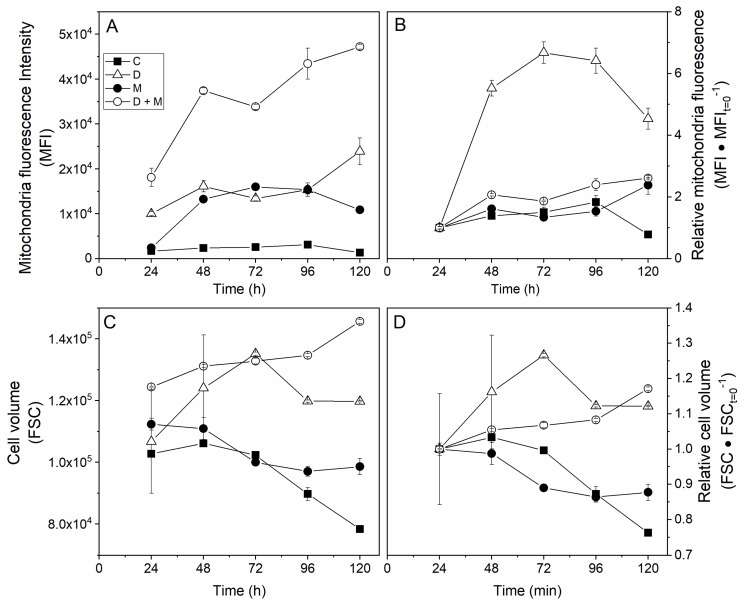
Long-term effects of adding hyperosmotic culture conditions combined with lipoic acid and hydroxycitrate drugs. (**A**) Mean fluorescence intensity (MFI) in a.u., (**B**) relative MFI monitoring, (**C**) cell volume (estimated by FSC in a.u.), and (**D**) relative cell volume for control (filled squares), drug (empty triangles), mannitol (filled circles) and both drug and mannitol (empty circles) conditions.

Cell adaptation to the drugs as well as a high osmotic environment was then characterized by first monitoring cell size and mitochondrial membrane potential (ΔΨm) during culturing with Rh123. In the control culture, the mean cell volume stayed quite constant from 24 to 72 h and then decreased linearly to diameters −12% (96 h) and −23% (120 h) lower than that measured at 48 h (Figure 4C,D). This cell volume decrease can be attributed to medium reduction of ionic and nutrient content, and thus of medium osmolarity, with cell uptake. Interestingly, cells cultured in 200 mM mannitol medium showed a mean volume increase of 16% at 72 h and 27% at 96 h and then decreased to a stable value. This result is opposite to the early event observations, but it suggests that cells rapidly react but do adapt, however, to this treatment. The addition of the drugs to the mannitol culture was found to limit cell size increase to 5–7% (72–120 h), while adding drugs to the control resulted in a lower cell volume at least 24 h earlier than observed in the control. The drugs may thus have an additive effect, causing cells to lower their volume, probably tampering with the advert effects of high osmotic conditions.

The long-term effect of growing cells in a 500 mOsm medium was a strong increase of the cell mitochondrial membrane potential, already reaching 7.5x at 72 h compared to the initial measurement made at 24 h, 9.1x at 96 h, and 8.6x at 120 h (Figure 4A,B). The culture with both mannitol and the drugs showed lower and quite constant values with 2.2x at 48 h, results that were similar to those for the control culture and the control with the drugs at 48 h with 1.7x and 1.8x the value, respectively. Indeed, although the mannitol culture had a highly specific behavior when looking at relative data, the high osmolarity condition still resulted in a higher total mitochondrial membrane potential per cell than the control. Moreover, and which was of interest, the drugs were found to cause an increase of the ΔΨm both in standard and high osmolarity conditions. The drugs may have thus succeeded in redirecting fluxes to the TCA, which is the major site of proton (H+) generation and storage at the mitochondrial intermembrane (IMM). Taken together, these results may suggest that either the drugs or the mannitol stimulated mitochondrial activity and, therefore, ATP turnover. However, since rhodamine 123 staining revealed ΔΨm and, indirectly, the H + gradient (ΔpH), we moved further with a complementary measurement enabling us to characterize the mitochondria oxidative pathways, such as OxPhos, with MitoTracker Red CM-H2XRos dye by estimating reactive oxygen species (ROS) production, which is known to be sensitive to mitochondria protonmotive force (Δp).

##### Cell Mitochondrial Activity

**Figure 5:**
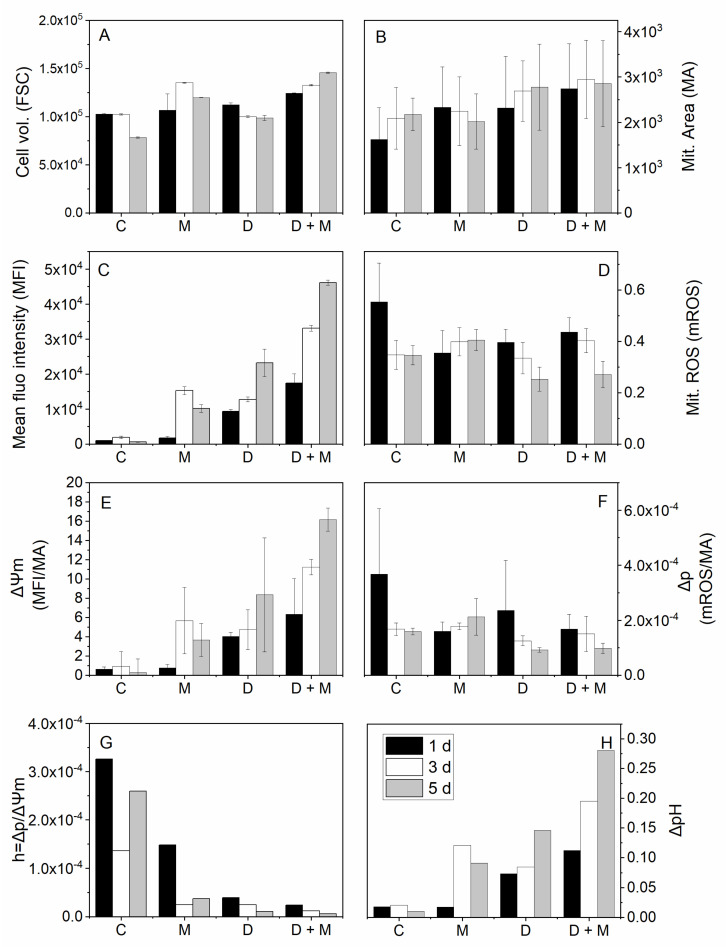
Mitochondrial activity and ROS generation in hyperosmotic culture conditions combined with lipoic acid and hydroxycitrate drugs (a.u.). C = control, M = mannitol condition, D = drugs condition, D + M = drugs and mannitol combination. Black bars are measurements of one day (1d) of culture, white bars are for three days (3d), and grey bars are for five days (5d) of culture. (**A**) Cell volume estimation based on forward scatter signal (FSC) parameter. (**B**) Mean mitochondria area, where determined, based on confocal microscopy images. (**C**) Mean fluorescence intensity (MFI) of rhodamine 123 used here to estimate mitochondrial membrane potential. (**D**) MitoTracker Red CM-H2XRos fluorescence intensity-reported mitochondrial ROS generation. (**E**) Mitochondrial mean fluorescence intensity normalized by mitochondria area ratio is intended to represent the mean mitochondrial membrane potential (ΔΨm). (**F**) Mitochondrial ROS (mROS) normalized by mitochondrial area (MA) roughly provides information on the mean mitochondrial membrane protonmotive force (Δp). (**G**) Ratio hypothesized as an estimation of mitochondria horsepower (h = Δp/ΔΨm). (**H**) Estimated mitochondrial proton gradient (ΔpH).

Cell behavior was studied focusing on specific cell stages, such as during the exponential growth phase on day 3 and the late exponential growth phase on day 5. The cell volume was stable in the control condition during the early exponential phase and decreased at day 5 (Figure 5A). It significantly increased in mannitol only (M) at day 3 (*p* ≤ 0.001) and day 5 (*p* ≤ 0.001) compared to control. Combing mannitol and drugs (D + M) also showed a significantly increased volume (*p* ≤ 0.001). The cell mitochondria area showed no significant increase in the control and the D culture with drugs only and it decreased with M and stayed constant in D + M (Figure 5B). Addition of drugs to mannitol condition resulted in a relative increase of mitochondria area but not significantly compared to mannitol. Rh123 staining revealed increased mitochondrial membrane potential (ΔΨm) with time in the M condition compared to control at day 3 and day 5 (*p* ≤ 0.01 and *p* ≤ 0.01, respectively) (Figure 5C). Similarly, the D condition led to a significant increase of ΔΨm with time (*p* ≤ 0.01). Interestingly, we report a cumulative effect on D + M conditions compared to control (*p* ≤ 0.001) and to M (*p* ≤ 0.001). ΔΨm normalized to mitochondria area showed the same trend with a cumulative effect of drugs addition as mannitol conditions and significantly increased at day 5 (*p* = 0.04) (Figure 5E).

Looking at the relative (to day 1) mitochondrial ROS fluorescence intensity, we see values that increased in the 200 mM mannitol culture while they significantly decreased in the cultures with the drugs from day 1 to day 5 (*p* ≤ 0.01) and in the combined drugs and mannitol culture from day 1 to day 5 (*p* ≤ 0.01) (Figure 5D). The same trend can be observed looking at the ratio of the mitochondria ROS intensity and the mitochondria area (Figure 5F), which we hypothetically assimilated to the mitochondria protonmotive force (Δp). These results suggest that the drugs effect prevails over that of mannitol in decreasing mitochondrial ROS production at day 5 (*p* ≤ 0.01).

Our results thus suggest a strong uncoupling in OxPhos, both in cultures with drugs and mannitol separately, compared to control and a cumulative effect of drugs added to mannitol conditions. The higher the uncoupling the more the protons, generated by the TCA reactions, accumulate at the mitochondrial intermembrane (IMM) because of a potential limitation rate of the respiratory chain, including proton leak reactions and inhibition of the “Phos” (ATP synthesis) counterpart of the OxPhos. Regarding glutaminolysis, proton congestion may suggest that the TCA was stimulated in those cells but with a repression or a limitation of mitochondrial respiration and complete carbon combustion, especially in the hyperosmotic condition. The drugs, which are known to force carbon fluxes towards the TCA, may have been successful in lowering ROS production, possibly by stimulating uncoupling proteins (UCPs) in both the D and D + M conditions.

The ratios presented on the histogram bars (Figure 5G,H) give some insight into the mitochondrial bioenergetic efficiency in the different cultures. Assuming that the mitochondria horsepower h may be defined as the Δp/ΔΨm ratio, these results show a clear decrease in mitochondrial efficiency under the M, D and D + M conditions (Figure 5G). Since Δp = ΔΨm − 59*ΔpH, the mitochondrial membrane potential seems to have increased contribution to the protonmotive force in the M and D conditions and had a cumulative effect in the D + M culture (Figure 5E). Then, Δp may have been restored by the higher proton gradient in hyperosmotic and drug conditions (Figure 5H). This supports the hypothesis of a significant decrease in the activity of ATP synthase.

## 3. Discussion

In this study, we targeted two enzymes known to be altered in cancer cells: pyruvate dehydrogenase (PDH), which is downregulated, and ATP citrate lyase (ACL), which is overexpressed. Alpha lipoic acid (ALA) is an inhibitor of pyruvate dehydrogenase kinase (PDK), a class of enzymes that inhibit pyruvate dehydrogenase (PDH). This means that pyruvate can no longer be converted to acetyl-CoA but passes through the lactate dehydrogenase (LDH) pathway. This is a mechanism that is very present in cancer cells and could, in part, explain the Warburg effect. Hydroxycitrate (HCA) is a known inhibitor of ACL. Based on previous work demonstrating that the combination of these drugs has antitumoral potential [50], we here investigated whether Chinese hamster ovary (CHO) cells behave as cancer cells. CHO cells were used to characterize the drug’s effects under inflammatory conditions, mimicked by increasing medium osmolarity with the addition of mannitol [8], known for not being metabolized by animal cells. It is now well-established that most cancers arise from inflammatory tissue [51,52,53] and the metabolic effects of inflammation are also well described [14].

The mitochondrial activity and redox balance are strongly dependent on ALA. It does not only have antioxidant properties but also acts as cofactor of many mitochondrial enzymes in addition to its action on PDH (Rochette et al., 2013). For instance, the regulation of complex I production of the superoxide anion through its interaction with 2-oxoglutarate dehydrogenase (Solmonson & DeBerardinis, 2018) can account in part for the restriction in ROS production (Figure 5D,F). To sum up, ALA and HCA combination is not efficient enough to manage TCA replenishment by reducing the Warburg effect (Figure 1D,E). Meanwhile, it positively regulates the mitochondria membrane electric potential (Figure 5C).

Tagaki et al. (2000) have already reported an increase of CHO cell diameter as well as increases of the specific glucose uptake rate and lactate production rate with the increase of the medium osmolarity [54]. Furthermore, they identified a critical (or threshold) osmolarity level at 450 mOsm for suspension cells. In our work assessing successive passages, we also noticed a clear effect of increasing from 400 mOsm to 500 mOsm on the cell-specific growth rate, while cells were found to recover the increase of their specific growth rate with successive passages (Figure S1). However, it is noteworthy that the increase of glycolysis and glutaminolysis seemed to change linearly with increasing osmolarity. Similarly, as in our study (Figure 4), they reported a cell volume increase with increasing osmolarity.

The important increase of glutaminolysis (i.e., decrease of Y_GLU/GLN_ ratio) with increasing osmolarity may have been a consequence of the glycolytic phenotype, with glutamine metabolized [27], feeding biochemical pathways leading to lactate production. We have previously demonstrated that glutamine normally contributes minimally to lactate production [55]. Therefore, it is thought that the increase of glutamine metabolized mainly contributes to feeding biochemical reactions of the TCA cycle, including those connected to anabolic reactions and cell respiratory machinery. This high glutaminolytic phenotype has been reported in various cancer cells and is thought to support energy production via the OxPhos metabolism [56].

This lower Y_GLU/GLN_ ratio simultaneous with a higher Y_LAC/GLC_ ratio suggests that the cells may compensate the loss of metabolic intermediates into lactate by increasing glutamine conversion to alpha-ketoglutarate. Then, TCA is replenished with citrate and acetyl-CoA intermediates, essential for lipid membrane synthesis of proliferating cells. Notably, in this study, the glutaminolytic phenotype correlated with cultures showing a high proportion of cells in the S phase, which has also been reported for human T lymphocytes [57].

To the best of our knowledge, we here describe for the first time how a high osmolarity condition causes an increase of the glutaminolytic phenotype, high-throughput glycolysis or the Warburg effect and decouples mitochondrial activity between carbon fluxes through the TCA, a major source of the proton gradient (ΔpH), and a reduced mitochondrial horsepower or ATP synthesis, such as reported in cancer cells [22,45].

## 4. Materials and Methods

### 4.1. Cell Line and Medium

The Chinese hamster cell line CHO-DXB11 was used in this study to assess the effect of high osmolarity and drugs combinations on cell growth and the metabolic responses. The culture medium used was CHO-SFM4, supplemented with glutamine and dextran sulfate to obtain final concentrations of, respectively, 4 mM and 0.05 mg/mL.

### 4.2. Cultures and Drug Treatments

Four different CHO cell cultures were performed in triplicate to detect the effects of D-mannitol, alpha-lipoic acid, and calcium hydroxycitrate on CHO cells. A first culture served as a control culture in which CHO cells were grown in culture medium without the addition of D-mannitol or a drugs cocktail, such as alpha-lipoic acid (ALA) and calcium hydroxycitrate (HCA). A second culture was supplemented with D-mannitol to study the osmotic effect of D-mannitol on the cells. A third culture consisted of CHO cells in culture medium with both ALA and HCA and the final culture was developed with both D-mannitol and the drugs. The four cultures were developed in shaken flask conditions and incubated in a Heracell 240i CO2 Incubator at 37 °C and 5% saturation in CO2 and under gentle agitation (120 rpm). From previous research, it was concluded that the effect of D-mannitol would be clearly visible at an applied concentration of 200 mM. As described by Schwartz et al. (2009), 8 μM ALA and 300 μM HCA results in a major cytotoxic effect of tumor cells with no demonstrable toxicity observed in healthy mice. Each flask of 125 mL, containing 25 mL of appropriate culture medium, whether supplemented with D-mannitol, the drugs or both, was inoculated with 2 × 10^5^ cells/mL. The four batch cultures were maintained for six days. Daily, 0.5 mL samples were taken from each culture to perform a cell count. The remaining samples were thereafter centrifuged at 1500 rpm (200 g) in a Thermo Micromax RF Centrifuge. The supernatants of each remaining sample were then collected in an Eppendorf tube and stocked at −30 °C. As well as the samples taken for the cell count, other samples were also taken daily to perform flow cytometry.

### 4.3. Analytical Methods

#### 4.3.1. Cell Count

A part of the 0.5 mL cell suspension taken daily was used to perform a cell count of the viable cells. Therefore, an appropriate dilution of both the cell suspension and trypan blue was undertaken. The membrane of dying or dead cells is porous and trypan blue is thus capable of entering those cells and coloring them. Viable cell counting was subsequently performed using a hemocytometer. The remaining cell suspension was centrifuged and stocked at −80 °C for further analysis of extracellular concentrations.

#### 4.3.2. Extracellular Metabolites Measurements

After six days of cell culture, the supernatants, stocked at −80 °C, were gradually thawed to determine extracellular concentrations of glucose, lactate, amino acids (including glutamine and glutamate) and antibodies. A YSI 2700 SELECT Bio-chemistry Analyzer was used to determine both glucose and lactate concentrations in mM. The extracellular concentrations of amino acids were determined using an Agilent 1290 HPLC system coupled to an Agilent 6460 triple quadruple mass spectrometer. Medium samples contained proteins which could precipitate during amino acids’ HPLC/MS/MS analyses and affect both the analyses and the results. The samples were thus deproteinated prior to sample injection by diluting them with 50% methanol to a ratio of 200 (0.005 mL sample in 0.955 mL of 50% methanol). After sample dilution, the samples were mixed with an internal standard buffer ISTD solution, which contained 50% of mobile phase A and 50% of mobile phase B to obtain the same buffer condition as the mobile phase. Samples were prepared the same day as the analysis since the acidic conditions lead to unstable components.

#### 4.3.3. Flow Cytometer and Microscopy Analysis

##### Sample Preparation

For microscopy analysis, the procedure was undertaken on days 1, 3, and 5 for each culture condition and its replicates. Following the cell count, 0.2 × 10^6^ to 1 × 10^6^ cells were collected and then centrifuged at 200× *g* for 5 min, the supernatant was discarded, and the cells were gently resuspended in prewarmed MT staining solution and incubated for 35 min under growth conditions. Thereafter, the cell suspension was centrifuged and resuspended in fresh and prewarmed phosphate saline buffer (PBS) to discard the remaining MT. After washing the cells with fresh PBS, the cells were centrifuged and resuspended in PBS containing 3.7% formaldehyde, freshly prepared and prewarmed, and subsequently incubated for 15 min under growth conditions. The cells were washed three times with PBS post-fixation. During cell fixation, DAPI staining solution was prepared by diluting the stock solution to 3 µM in staining buffer (100 mM Tris, pH 7.4, 150 mM NaCl, 1 mM CaCl_2_, 0.5 mM MgCl2, 0.1% IGEPAL^®^CA-630). After the PBS washes the cell pellet was resuspended in DAPI staining solution and incubated for 15 min at room temperature, then the staining solution was removed, and the cell pellet resuspended in PBS for fluorescence microscopy. For flow cytometry analysis, a similar number of cells were collected but they were fixed immediately. They were then DAPI stained with the same procedure described above but left in the staining solution for direct flow cytometry analysis every day of the culture period.

##### Flow Cytometer

After the 15 min incubation at room temperature, DNA content analysis was undertaken as soon as possible for every day of the culture with a BD FACS Canto II (BD Biosciences, Franklin Lakes, NY, USA), using a violet laser with a 405 nm wavelength, a 450/50 bandpass filter, and the appropriate laser properties set. The samples were passed with a low flow to ensure the correct identification of DNA content for posterior analysis by FlowJo^®^ v10 (FlowJo LLC, Ashland, OR, USA) to give the percentage of average DNA content in the populations at different times of the cultures.

##### Mitochondrial Membrane Potential Measurement

A total of 5 × 10^5^ live cells were taken daily from each flask and centrifuged for 5 min at 200× *g*. Cells were resuspended in 300 μL of culture medium containing 10 μg/mL rhodamine 123 (membrane potential; Invitrogen, ref. R302), incubated in the dark for 30 min, then washed twice each for 15 min in 300 μL of phosphate-buffered saline (PBS).

##### Fluorescence Microscopy

Two different probes were used, reduced MitoTracker Red (MT) and 4′,6-diamidino-2-phenylindole (DAPI) dilactate, both from ThermoFisher, Waltham, MA, USA, to assess mitochondrial activity and DNA content, respectively, in the different culture conditions. The staining solution of MT was prepared fresh every two days by adding DMSO to a final concentration of 1 mM and then diluting it 10 times into an intermediate solution, with a subsequent final dilution to a 900 nM staining concentration, as advised by the manuals and protocols of the provider. The DAPI Dilactate stocks and solutions were made according to provider specifications.

Following the staining of both probes the cells were mounted on a glass plate and then observed through a Zeiss Axio Observe Z1 Inverted Fluorescence Microscope (Carl Zeiss Canada, Toronto, ON, Canada). The aperture and condenser settings were maintained throughout the analysis of all culture samples to ensure homogeneity. The lasers employed were 350 and 595 nm for the excitation of DAPI and MT, respectively. After the photos were taken, a quantitative analysis of the images was performed using the open source software CellProfiler v3.0.0. The analysis through this software consisted of identifying the objects of interest, the nuclei and the MT-labeled mitochondria, and quantifying the differences of emission intensity linked to mitochondrial activity and DNA content.

### 4.4. Statistical Analysis

Data are presented as mean ± the standard error of the mean (*n* = 3). Statistical analysis was performed with Origin software (version 2018). Values of *p* < 0.05 were considered significant and the notations of * (*p* < 0.05), ** (*p* ≤ 0.01), and *** (*p* ≤ 0.001) were used for the comparison versus the control group by Student’s t-test.

## 5. Conclusions

We report that hyperosmolar conditions triggered high-throughput glycolysis and enhanced glutaminolysis in CHO cells, such as during cancer cell proliferation. Quite interestingly, and inversely to cancer cells, CHO cells showed an increase in mitochondrial membrane potential (ΔΨm) but with a probably reduced mitochondrial horsepower (h = Δp/ΔΨm) under perturbed conditions (hyperosmolarity and drugs). This may be explained as an evolutionary strategy of mitochondria used to maintain a stable protonmotive force (Δp) through enhanced hyperfusion. Additional studies on cancer cells are needed to better understand the metabolic peculiarities of cancer cells, which are probably not able to maintain the mitochondria protonmotive force.

## Data Availability

Data are available upon request to the corresponding author.

## Supplementary Materials

The following are available online at https://www.mdpi.com/article/10.3390/metabo11060344/s1, Figure S1: CHO cells behavior along successive passages under high osmolarity conditions, Figure S2: Effects of hyperosmotic culture conditions combined with lipoic acid and hydroxycitrate drugs, Figure S3: Metabolites variation in different batch cultures.
