# Supplementary material for: Hyperosmolarity Triggers the Warburg Effect in Chinese Hamster Ovary Cells and Reveals a Reduced Mitochondria Horsepower
Source: Metabolites. 2021 May 26;11(6):344. doi: 10.3390/metabo11060344 (PMC8226498; doi:10.3390/metabo11060344)
Supplement: Supplementary file 1 [file metabolites-11-00344-s001.zip › metabolites-1213974-supplementary.pdf]

metabolites

# Supplementary Materials

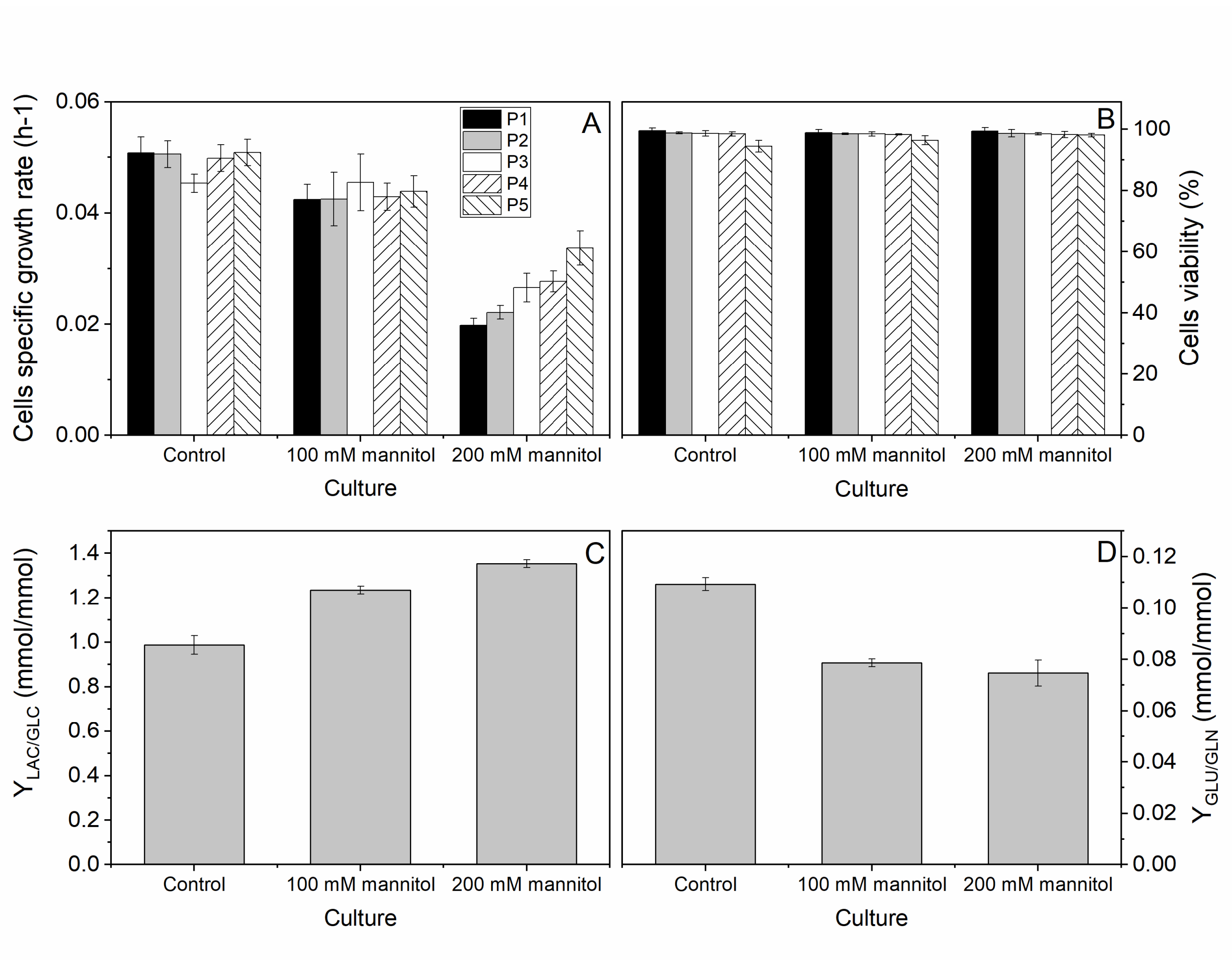


**Figure S1.** CHO cells behavior along successive passages under high osmolarity conditions. (**A**) Cells specific growth rates in control and high osmolarity (100 mM and 200 mM mannitol) conditions along successive passages (P1-5). Growth rates were more affected under 200 mM mannitol compared to other conditions. (**B**) Cell viability remained flat for all conditions along P1-5. (**C**) Mean lactate yields of the five passages per mole of consumed glucose ($Y_{LAC/GLC}$) were reported better in high osmolarity conditions. (**D**) Conversely, mean glutamate yield per mole of consumed glutamine were lower in high osmolarity conditions.

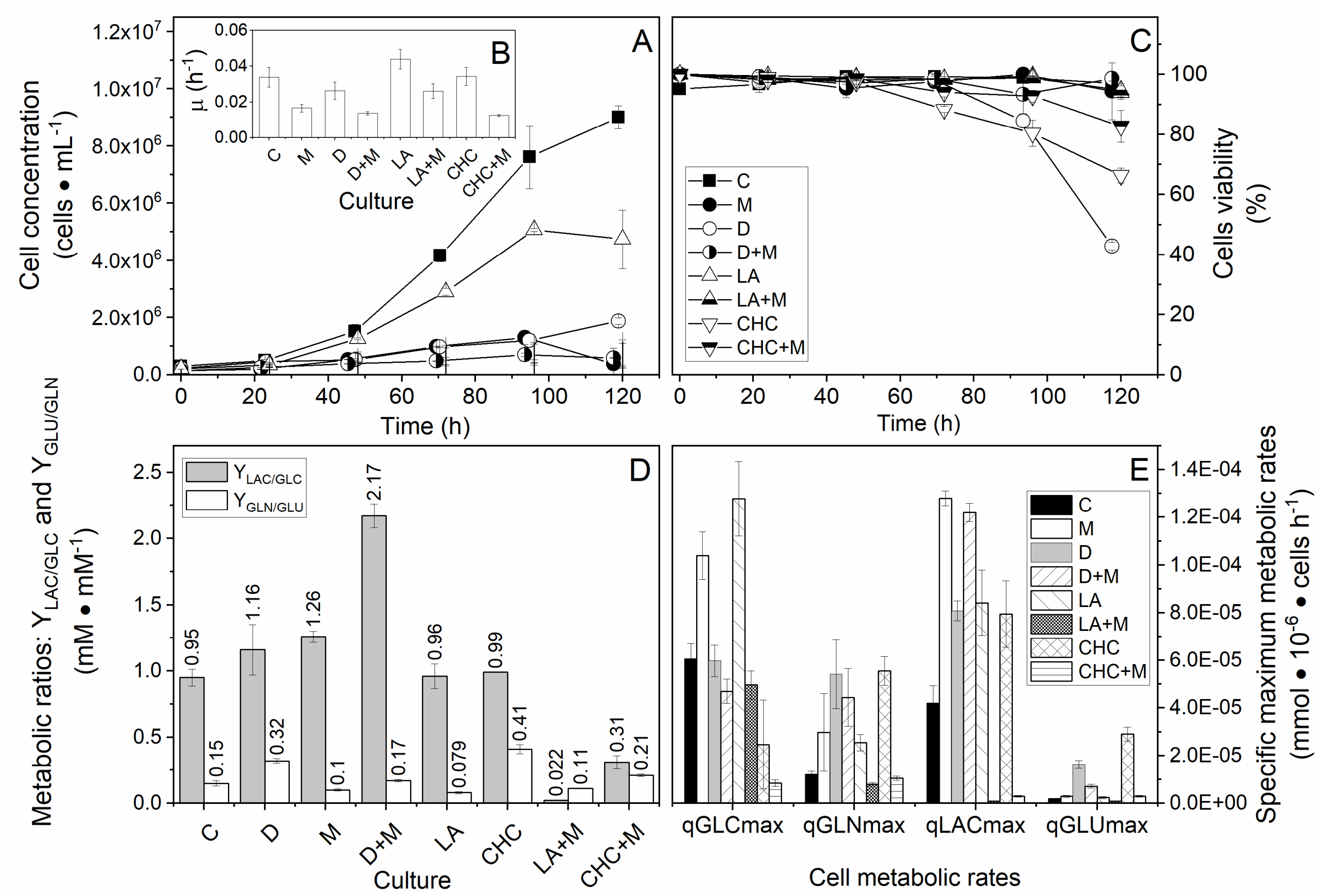


**Figure S2.** Effects of hyperosmotic culture conditions combined with lipoic acid and hydroxycitrate drugs. C = control, M = mannitol, D = drugs combination, D+M = drugs and M culture. LA = alpha-lipoic acid, LA+M = alpha-lipoic acid and mannitol, HCA = hydroxycitrate, HCA+M = hydroxycitrate and mannitol.

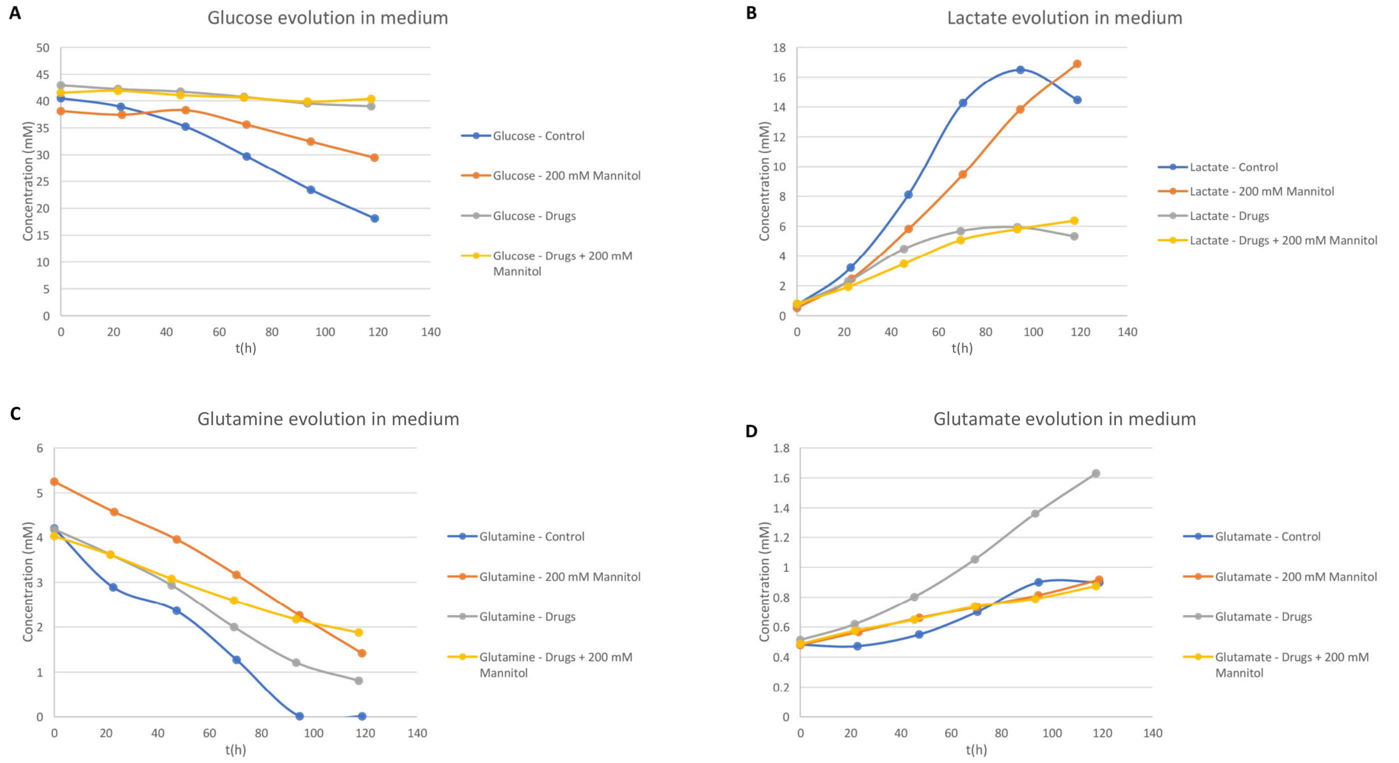


**Figure S3.** Metabolites variation in different batch cultures. (**A**) Glucose consumption is highly inhibited under drug conditions (D and D+Mannitol) compared to the control condition or under mannitol alone. (**B**) Lactate production is correlated with glucose consumption for each condition. (**C**) Glutamine is globally less consumed under disturbed conditions. It is noted that it is more consumed in drug conditions compared to Mannitol. (**D**) Glutamate, which is mainly derived from the consumption of glutamine, is significantly more important in drug conditions. This is a marker of saturation of the glutaminolysis pathway in drug conditions.
